# Supplementary material for: URI alleviates tyrosine kinase inhibitors-induced ferroptosis by reprogramming lipid metabolism in p53 wild-type liver cancers
Source: Nat Commun. 2023 Oct 7;14:6269. doi: 10.1038/s41467-023-41852-z (PMC10560259; doi:10.1038/s41467-023-41852-z)
Supplement: Supplementary file 1 — Supplementary Information [file 41467_2023_41852_MOESM1_ESM.pdf]

Supplementary Information

URI alleviates tyrosine kinase inhibitors-induced ferroptosis by reprogramming lipid metabolism in p53 wild-type liver cancers

Zhiwen Ding, Yufei Pan, Taiyu Shang, Tianyi Jiang, Yunkai Lin, Chun Yang, Shujie Pang, Xiaowen Cui, Yixiu Wang, Xiao fan Feng, Mengyou Xu, Mengmiao Pei, Yibin Chen, Xin Li, Jin Ding, Yexiong Tan, Hongyang Wang, Liwei Dong, Lu Wang.

Supplementary Figure 1

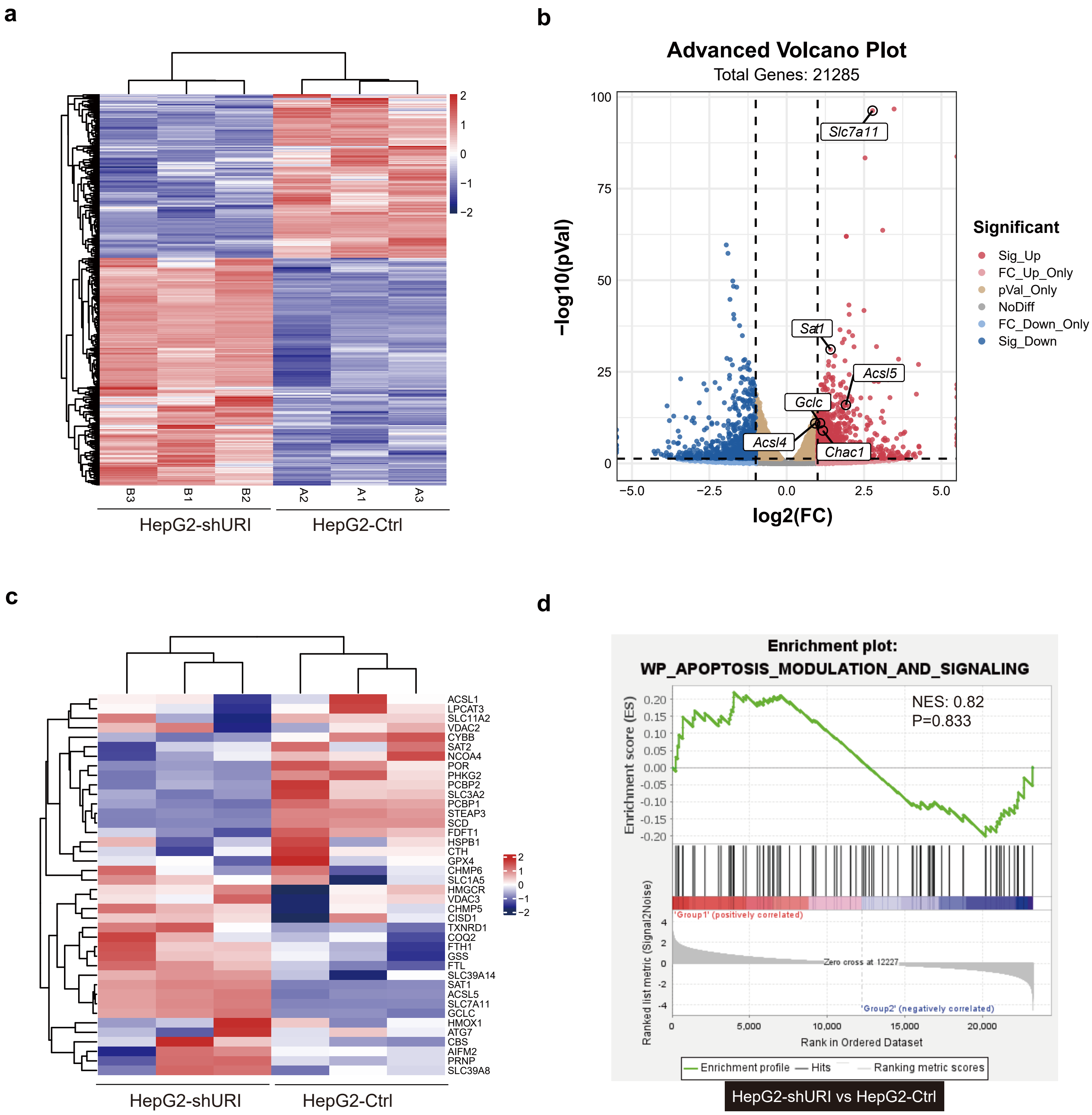

Supplementary Fig. 1: RNA-sequencing analysis of URI depletion in HepG2 cells. a, Heatmap summarized RNA-seq data for HepG2-Ctrl and HepG2-shURI cells with 975 up-regulated genes and 701 downregulated genes (n=3 biological replicates). **b,** Volcano plot of gene expression in HepG2-shURI vs HepG2-Ctrl cell. The fold change is the average gene expression of the shURI group vs Ctrl group. Differentially expressed genes are shown in red (upregulated) and blue (downregulated). **c,** Heatmap summarized mRNA transcripts of ferroptosis-related genes for HepG2-Ctrl and HepG2-shURI cells. **d,** GSEA indicates that WP 'Apoptosis modulation and signaling' was not enriched in the HepG2-shURI cells based on additional gene alterations, the FDR *P*-value and NES were determined by GSEA software (v4.1.0). Data are means ± SEM. Statistical analysis of RNA-seq data is performed using edgeR and *P*-values are FDR-adjusted. Source data are provided as a Source Data file.

Supplementary Figure 2

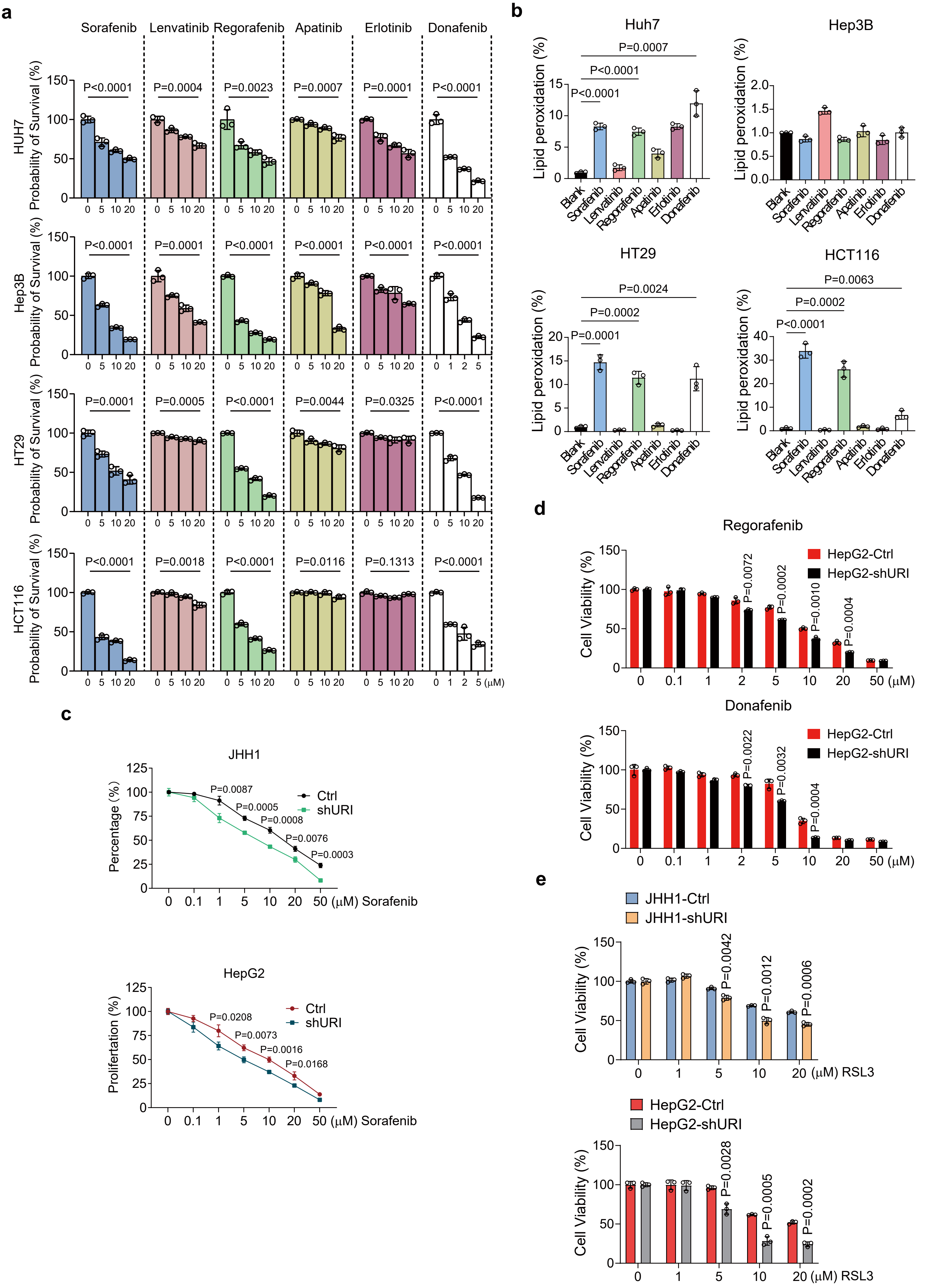

Supplementary Fig. 2: URI depletion promotes TKI-induced ferroptosis in cancer cells. a, Relative viability of HUH7, Hep3B, HT29 and HCT116 cells treated with different concentrations of sorafenib, lenvatinib, regarofenib, apatinib, erlotinib or donafenib for 48 h, and cell viability was measured (n = 3 biological replicates). **b,** HUH7, Hep3B, HT29 and HCT116 cells were treated as indicated. Sorafenib, 10 μM; lenvatinib, 10 μM for Hep3B cells and 20 μM for HUH7, HT29 and HCT116 cells; regarofenib 10 μM; apatinib 20 μM; erlotnib 20 μM; donafenib 10 μM for HUH7 and HT29 cells, 5 μM for Hep3B and HCT116 cells. Lipid peroxidation was measured by C11-BIODY staining (n = 3 biological replicates). **c,** Cell viablility after 48 h of sorafenib treatment was assessed by CCK-8 (n = 3 biological replicates). **d,** Relative viability of HepG2-Ctrl and HepG2-shURI cells treated with different concentrations of regarofenib or donafenib for 48 h and cell viability was measured (n = 3 biological replicates). **e,** Relative viability of JHH1 cells and HepG2 cells with or without URI depletion treated with different concentrations of RSL3 for 48 h and cell viability was measured (n = 3 biological replicates). Data are means ± SEM. Statistical significance in **a-e** is determined by two-tailed unpaired *t*-test. Source data are provided as a Source Data file.

Supplementary Figure 3

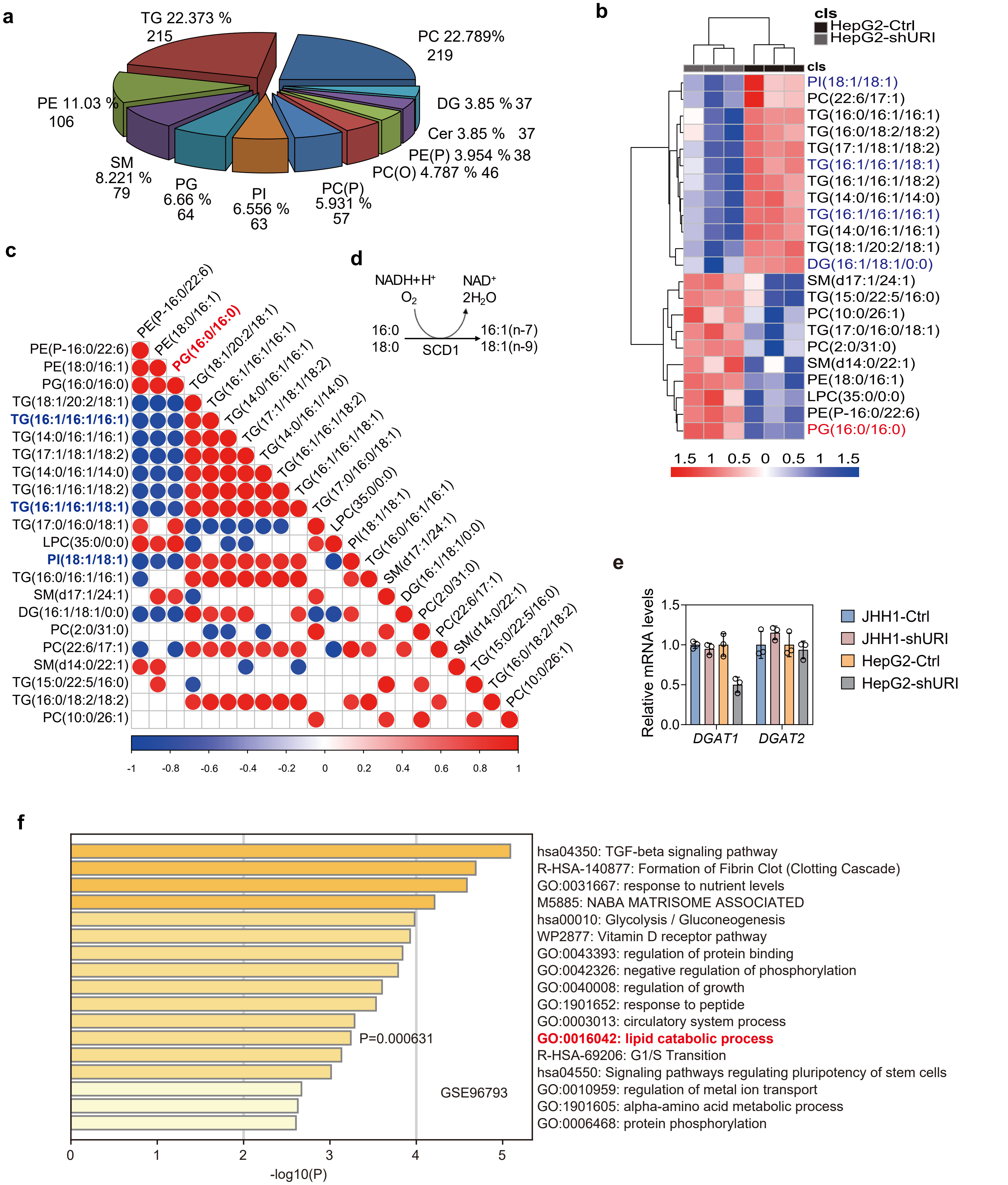

Supplementary Fig. 3: URI depletion reprogrammed lipid metabolism of tumor cells. a, Composition of lipid classes in HepG2-Ctrl and HepG2-shURI cells detected by liquid chromatography–mass spectrometry/mass spectrometry. **b,** Heatmap showed fold-changes of lipid species between HepG2-Ctrl and HepG2-shURI cells. **c,** Correlation heatmap of different lipid species based on the basis of **(b)**. **d,** Schematic of the reaction catalysed by SCD1. **e,** Relative mRNA expression of lipogenic genes *DGAT1* and *DGAT2* in HepG2 cells with or without URI depletion (n = 3 biological replicates). **f,** GO enrichment analysis of GSE96793 and the altered pathways after sorafenib treatment were listed. Data are means ± SEM. Statistical significance in **e** is determined by two-tailed unpaired *t*-test. Source data are provided as a Source Data file.

Supplementary Figure 4

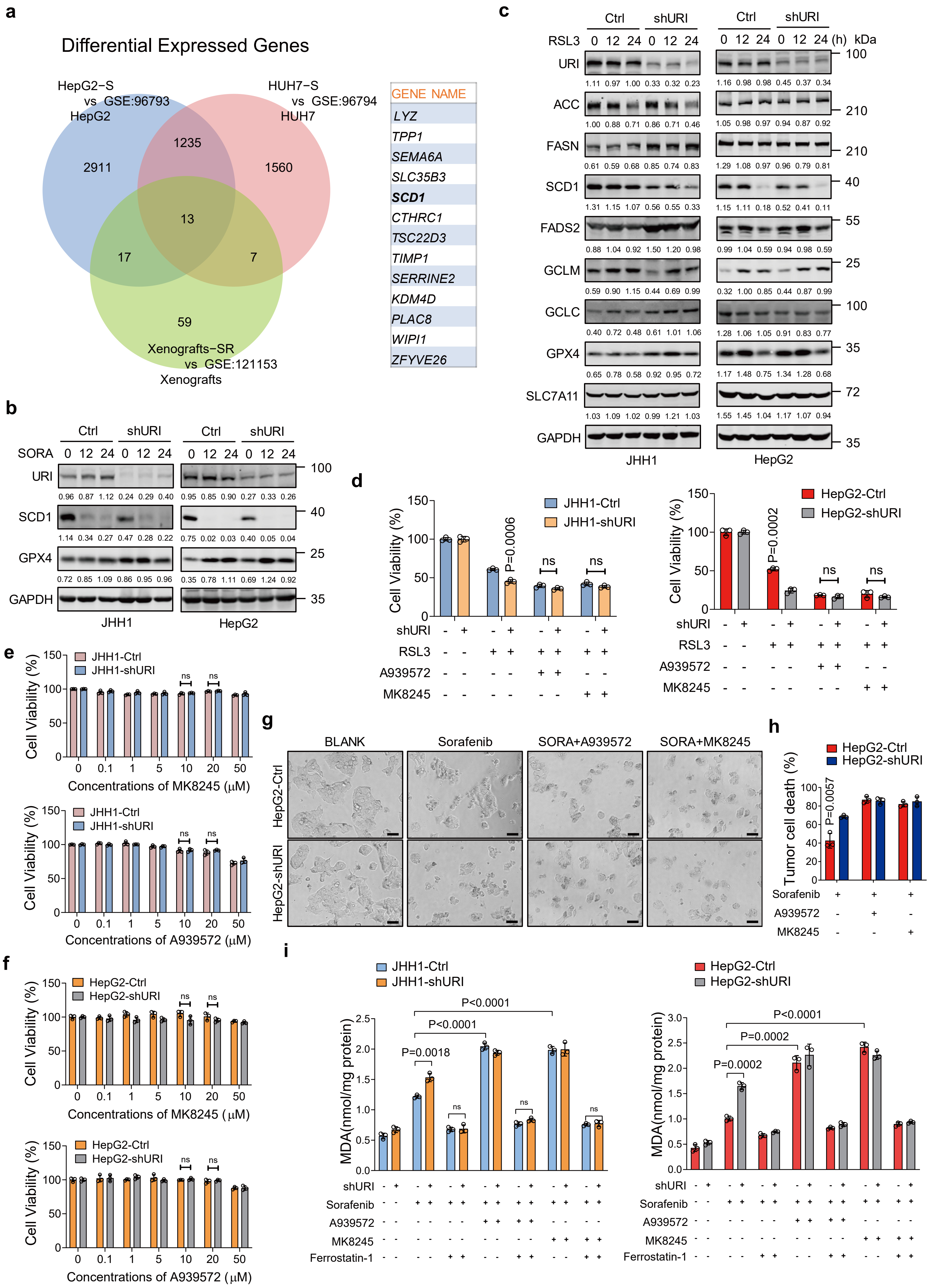

Supplementary Fig. 4: URI alleviated TKI-induced ferroptosis in a SCD1-dependent manner. a, Venn diagram showed overlap of the 13 genes significantly altered in the indicated GEO dataset after administrating with sorafenib in HepG2, HUH7 cells and Xenografts mouse models. **b,** Cells were treated with 20μM sorafenib for indicated time points, and cell lysates were immunoblotted with indicated antibodies. **c,** Cells were treated with 20μM RSL3 for indicated time points, and cell lysates were immunoblotted with indicated antibodies. **d,** Relative viability of JHH1 and HepG2 cells with or without URI depletion treated with 10μM RSL3 in the presence and absence of A939572 (10μM) or MK8245 (10μM) for 48 h. Cell viability was measured (n = 3 biological replicates). **e, f,** Relative viability of JHH1 and HepG2 cells with or without URI depletion treated with A939572 or MK8245 at the indicated concentrations for 48 h. Cell viability was measured (n = 3 biological replicates). **g,** Representative images of HepG2-Ctrl and HepG2-shURI cells cultured with 10μM sorafenib in the presence and absence of A939572 (10μM) or MK8245 (10μM) for 48 h (n = 3 biological replicates). Scale bar, 50 μm. **h,** Cells in (**g**) were stained with PI followed by flow cytometry. Quantification of three independent assays (n = 3 biological replicates per assay). **i,** MDA assay of JHH1 and HepG2 cells with or without URI depletion treated with 10μM sorafenib in the presence and absence of A939572 (10μM), MK8245 (10μM), or ferrostatin-1 (20μM) for 48 h (n = 3 biological replicates). All blots were the representative images from 3 independent experiments. Data are means ± SEM. Statistical significance in **d-f, h** and **i** is determined by two-tailed unpaired *t*-test. Source data are provided as a Source Data file.

Supplementary Figure 5

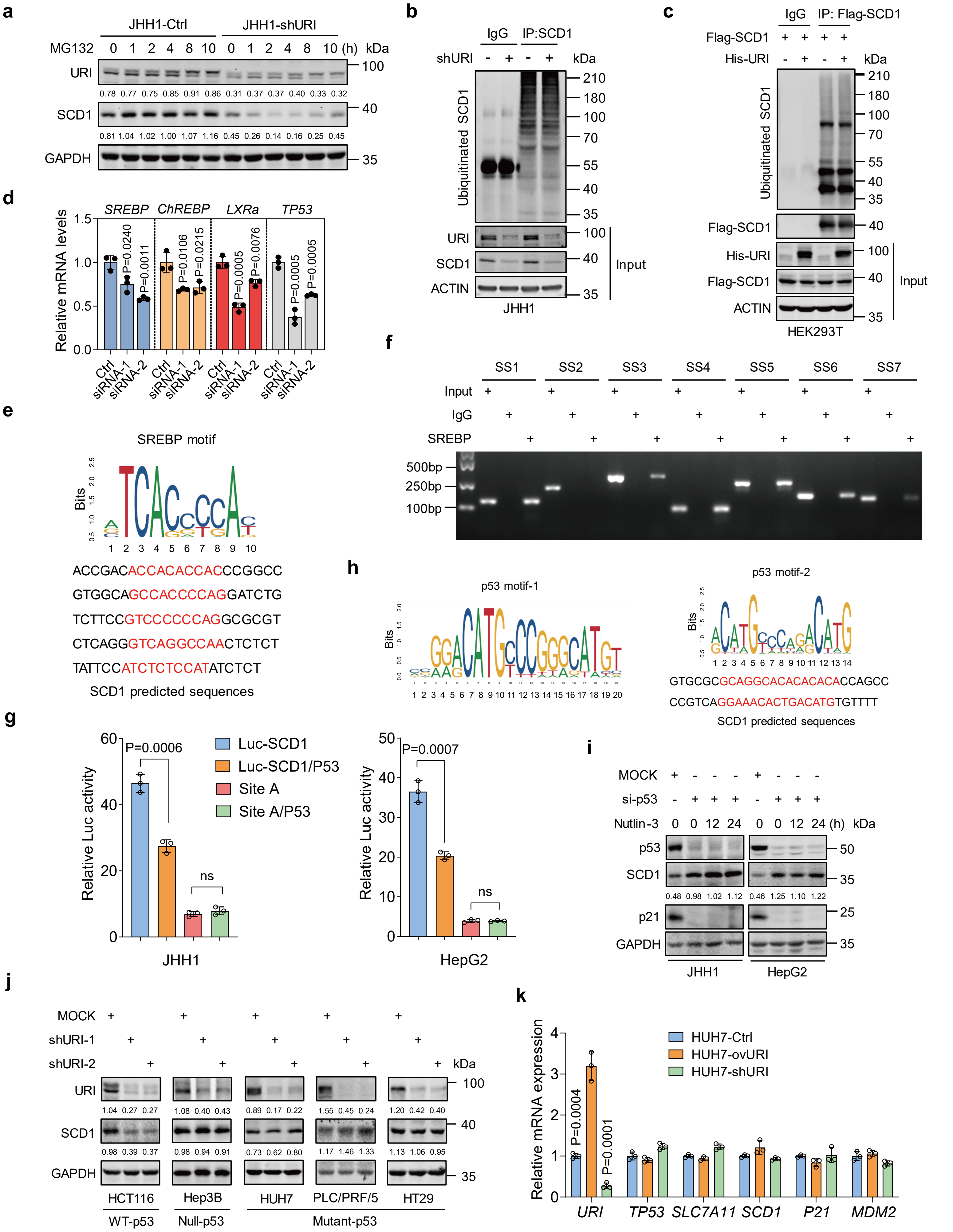

Supplementary Fig. 5: URI promotes SCD1 transcription by inhibiting p53. a, JHH1-Ctrl and JHH1-shURI cells were treated with 10μM MG132 at the indicated times. Cell lysates were immunoblotted with indicated antibodies. **b,** JHH1-Ctrl and JHH1-shURI cells were transfected with HA-tagged ubiquitin for 48 h before anti-SCD1 immunoprecipitation and immunoblotting was performed. **c,** HEK293T cells with or without stably His-URI expressing were transfected with HA-tagged ubiquitin and Flag-SCD1 plasmids for 48 h before anti-Flag immunoprecipitation and immunoblotting was performed. **d,** Relative mRNA expression of *SREBP*, *ChREBP*, *LXRα* and *TP53* in JHH1 cells transfected with siRNA of the indicated genes (n = 3 biological replicates). **e,** Sequence logo for SREBF1 binding. **f,** ChIP assay was carried out in HepG2 cells and specific primers flanking the SREBP binding sites (SS1-SS7) were used (n = 3 biological replicates). **g,** JHH1 and HepG2 cells were transfected with pGL3 reporter plasmids containing *SCD1* promoter (Luc-SCD1) or the sequence of peak site A (Chr10: 100347233-100364826) in the gene body (Site A) as shown in **Fig. 4e**, the cells were cotransfected with p53-expressing or control plasmid. Luciferase activity was measured (n = 3 biological replicates). **h,** Sequence logo for p53 binding. **i,** JHH1 and HepG2 cells with p53 knockdown were treated with 10mM nutlin-3 at the indicated times, and cell lysates were immunoblotted with indicated antibodies (n = 3 replicates). **j,** Colon cancer cells (HCT116, HT29) and liver cancer cells (Hep3B, HUH7 and MHCC97H) with different p53 status were infected with lentivirus containing different shURI oligos, then cell lysates were immunoblotted with indicated antibodies. **k,** Relative mRNA expression of p53 targeted genes in cells (n = 3 biological replicates). All blots were the representative images from 3 independent experiments. The values under the WB panels indicate the quantification of the intensities normalized to GAPDH or ACTIN in each experiment. Data are means ± SEM. Statistical significance in **d**, **g** and **k** is determined by two-tailed unpaired t-test. Source data are provided as a Source Data file.

Supplementary Figure 6

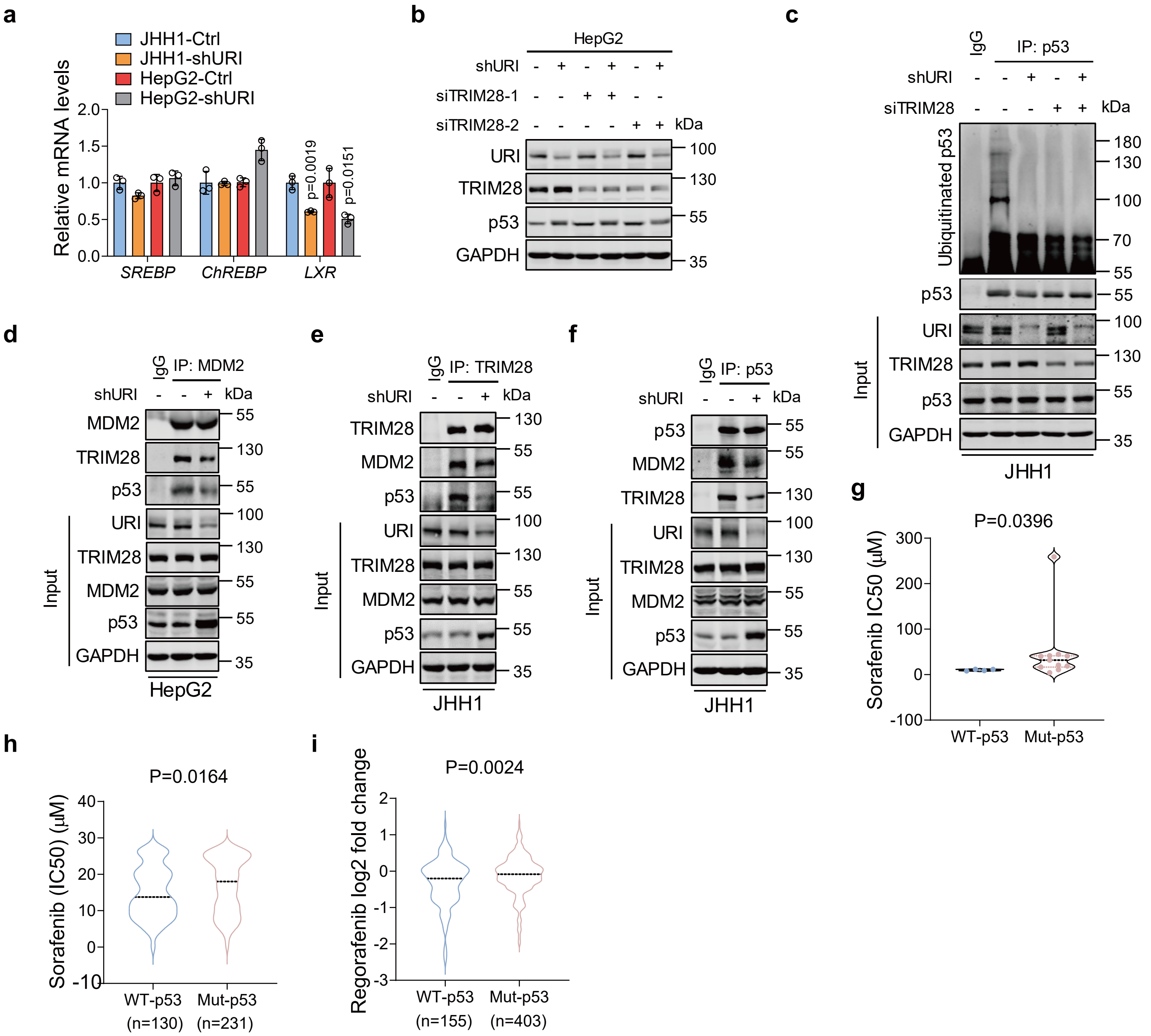

Supplementary Fig. 6: URI promotes p53 ubiquitination and degradation by interacting with TRIM28. a, Relative mRNA expression of *SREBP*, *ChREBP* and *LXRα* in JHH1 and HepG2 cells with or without URI depletion (n = 3 biological replicates). **b,** HepG2-Ctrl and HepG2-shURI cells were transfected with MOCK or siTRIM28 for 48 h and cell lysates were collected and immunoblotted with indicated antibodies. **c,** JHH1-Ctrl and JHH1-shURI cells transfected with HA-tagged ubiquitin and together with si-TRIM28 or scramble siRNA for 48 h, then cells were treated with 10μM MG132 for 6 h before anti-p53 immunoprecipitation and immunoblotting were performed. **d,** HepG2-Ctrl and HepG2-shURI cell lysates were subjected to anti-MDM2 immunoprecipitation and then immunoblotted with indicated antibodies. **e,** JHH1-Ctrl and JHH1-shURI cell lysates were subjected to anti-TRIM28 immunoprecipitation and then immunoblotted with indicated antibodies. **f,** JHH1-Ctrl and JHH1-shURI cell lysates were subjected to anti-p53 immunoprecipitation and then immunoblotted with indicated antibodies. **g,** Sorafenib IC50 values of liver cancer cell lines with different p53 status according to CCLE datasets[41]. Each dot represents a cell line. **h,** Sorafenib IC50 values of p53 wild-type cell lines (n=130) compared with p53 mutant cell lines (n=231). Data were adapted from CCLE database. **i,** Efficacy of regorafenib in p53 wild-type cell lines (n=155) compared with p53 mutant cell lines (n=403) according to CCLE database. All blots were the representative images from 3 independent experiments. Data are means ± SEM. Statistical significance is determined by two-tailed unpaired *t*-test in **a** and two-tailed Mann-Whitney test in **g-i**. Source data are provided as a Source Data file.

Supplementary Figure 7

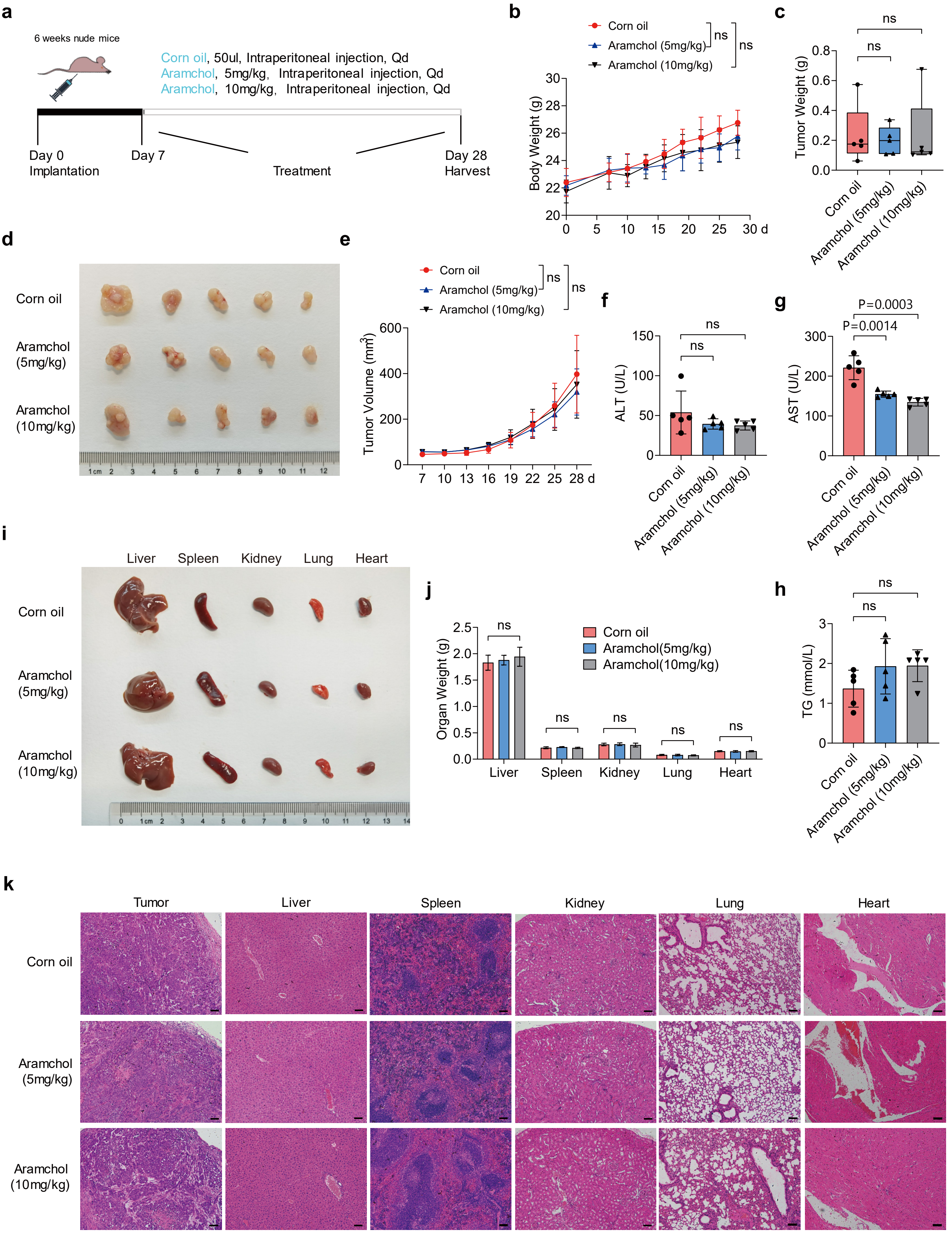

Supplementary Fig. 7: Safety of aramchol in xenografts mice models. a, Schematic representation of the administration schedule for aramchol. **b,** Time course of body weight of nude mice in the xenograft models treated aramchol with the indicated concentrations (n=5 mice per group). **c,** Tumor weight of each group of HepG2 xenografts at the end of treatment is plotted, boxplot center line, mean; box limits, upper and lower quartile; whiskers min. to max (n=5 mice per group). **d,** Representative tumor images of each group of HepG2 xenografts at the end of treatment (n=5 mice per group). **e,** Tumor growth curves of HepG2 xenografts (n=5 mice per group). **f, g,** ALT (**f**) and AST (**g**) levels in each group were measured. (n=5 mice per group). **h,** TG levels in each group were measured (n=5 mice per group). **i,** Representative images of liver, spleen, right kidney, left lung and heart from treated nude mice. **j,** The weight of liver, spleen, right kidney, left lung and heart in each group were measured (n=5 mice per group). **k,** Representative hematoxylin and eosin (H&E) staining of tumors, livers, spleens, right kidneys, left lungs and hearts from nude mice. (n=5 mice per group) Scale bar, 100 μm. Data are means ± SEM. Statistical significance is determined by two-way ANOVA with Tukey multiple comparisons test in **b**, **e** and two-tailed unpaired t-test in **c, f-h** and **j**. Source data are provided as a Source Data file.

Supplementary Figure 8

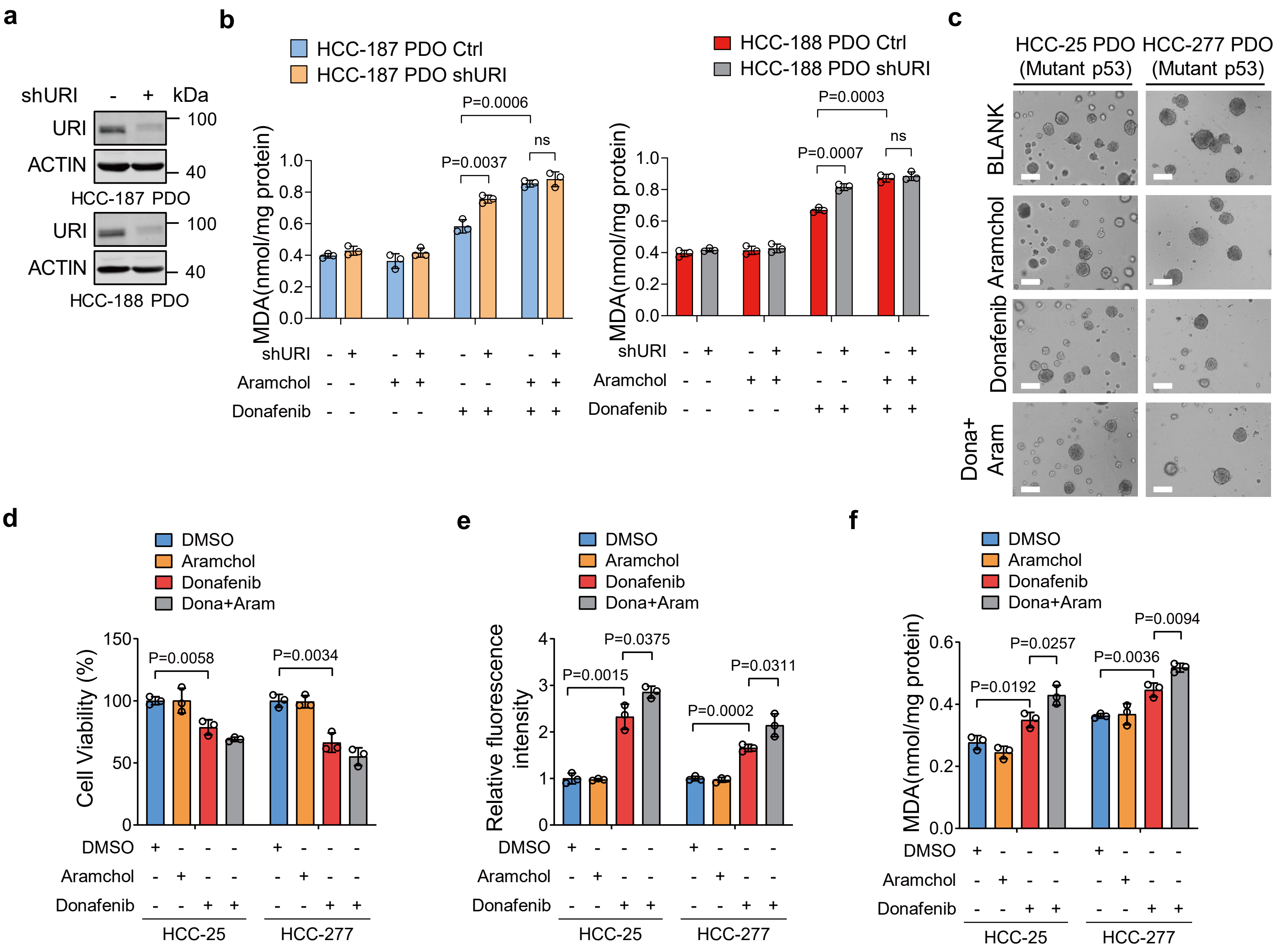

Supplementary Fig. 8: The therapeutic effect of the combination therapy in patient-derived organoids. a, HCC187 PDOs and HCC188 PDOs were infected with shURI lentivirus, and cell lysates were immunoblotted with indicated antibodies. **b,** MDA assay of HCC-187 PDOs and HCC-188 PDOs with or without URI depletion treated with donafenib (10μM) in the presence or absence of aramchol (10μM) for 5 days (n = 3 biological replicates). **c,** Representative images of HCC-25 PDOs and HCC-277 PDOs (both with p53 mutation) treated with aramchol, donafenib or combination therapy for 5 days. Scale bar: 100 μm. **d,** Relative cell viability of HCC-25 PDOs and HCC-277 PDOs treated with donafenib (10μM) in the presence or absence of aramchol (10μM) for 5 days (n = 3 biological replicates). **e,** Liperfluo assay of HCC-25 PDOs and HCC-277 PDOs treated with donafenib (10μM) in the presence or absence of aramchol (10μM) for 5 days (n = 3 biological replicates). **f,** MDA assay of HCC-25 PDOs and HCC-277 PDOs treated with donafenib (10μM) in the presence or absence of aramchol (10μM) for 5 days (n = 3 biological replicates). Data are means ± SEM. Statistical significance in **b** and **d-f** is determined by two-tailed unpaired *t*-test. Source data are provided as a Source Data file.

Supplementary Figure 9

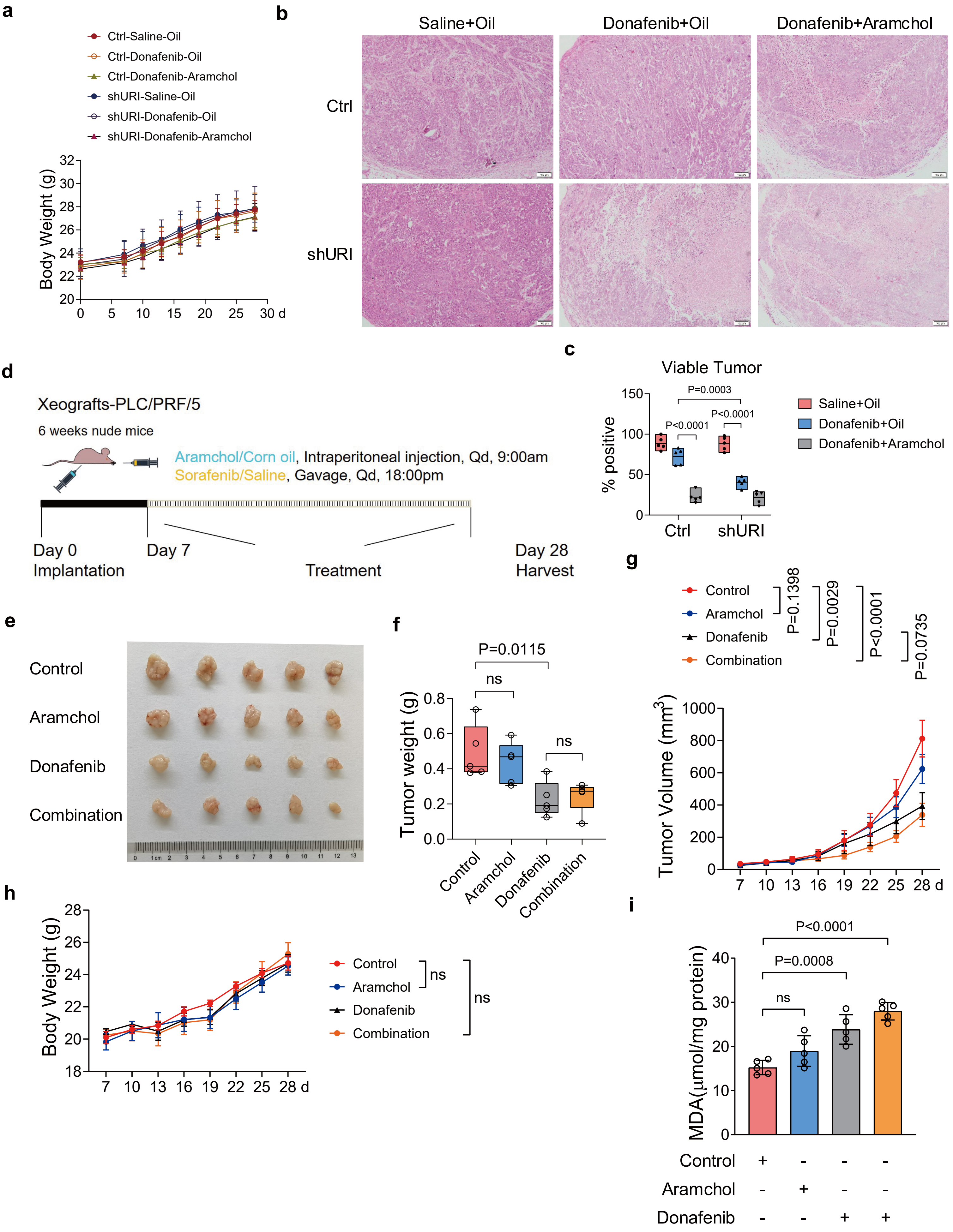

Supplementary Fig. 9: The therapeutic effect of the combination therapy *in vivo*. a, Time course of body weight of nude mice in the xenograft models with donafenib or combination therapy (n = 5 mice per group). **b,** Representative hematoxylin and eosin (H&E) staining of tumors from nude mice treated with donafenib or combination therapy (n = 5 mice per group). Scale bar, 100 μm. **c,** Tumor viability assessed in H&E slides from the xenografts models is plotted, boxplot center line, mean; box limits, upper and lower quartile; whiskers min. to max. (n = 5 mice per group). **d, e,** Schematic representation of the therapy schedule for donafenib or combination therapy in PLC/PRF/5 xenografts in **d**, and representative tumor images of each group of PLC/PRF/5 xenografts at the end of treatment was shown in **e** (n=5 mice per group). **f,** Tumor weight of each group of PLC/PRF/5 xenografts at the end of treatment is plotted, boxplot center line, mean; box limits, upper and lower quartile; whiskers min. to max (n = 5 mice per group). **g,** Growth curves of each group of PLC/PRF/5 xenografts (n=5 mice per group). **h,** Body weight of each group of PLC/PRF/5 xenografts at the end of treatment (n=5 mice per group). **i,** MDA assay of each group of PLC/PRF/5 xenografts at the end of treatment (n=5 mice per group). Data are means ± SEM. Statistical significance is determined by two-way ANOVA with Tukey multiple comparisons test in **a, g** and **h** and two-tailed unpaired t-test in **c, f** and **i**. Source data are provided as a Source Data file.

Supplementary Figure 10

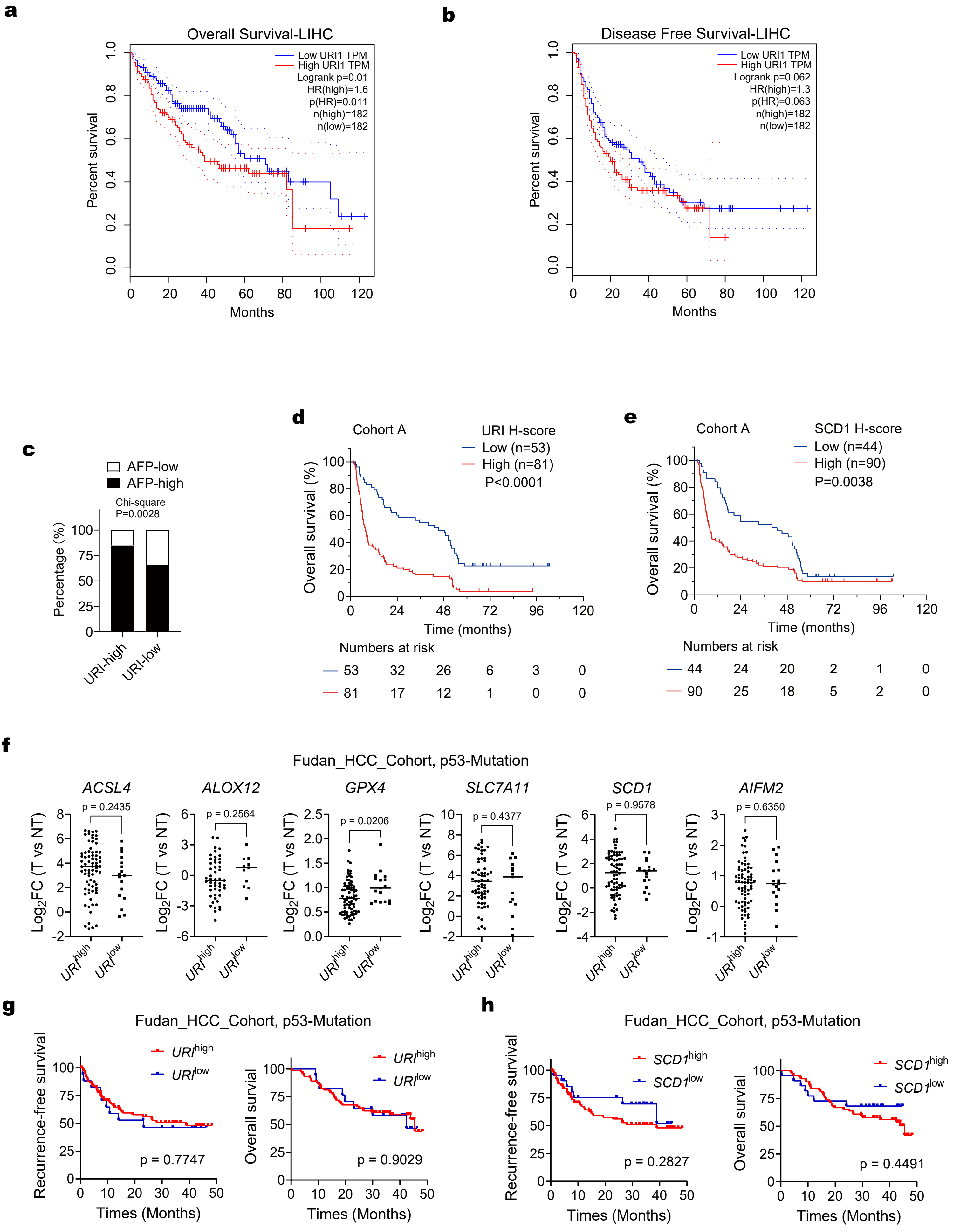

Supplementary Fig. 10: URI is associated with poor survival in HCC patients with wild-type p53. a, Kaplan-Meier curves for OS between *URI* high expression and *URI* low patients in liver hepatocellular carcinoma (LIHC) (n=364 patients). Data were adapted from GEPIA[44] (http://gepia.cancer-pku.cn/). **b,** Kaplan-Meier curves for DFS between *URI* high and *URI* low patients in liver hepatocellular carcinoma (LIHC) (n=364 patients). Data were adapted from GEPIA (http://gepia.cancer-pku.cn/). **c,** Correlation between AFP levels and URI expression in HCC tissues from cohort A was evaluated using a chi-square test (n=134 patients). **d, e,** Kaplan–Meier survival analysis of cohort A stratified by levels of URI (**d**) and SCD1 (**e**) (n=134 patients). Numbers below plots represent numbers of patients. **f,** The transcriptional status of certain genes between *URI*^{high} versus *URI*^{low} patients with mutant p53 from Fudan_HCC_Cohort， the transcriptional levels were showed as log2 values of gene FPKM ratios between tumor (T) and paired non-tumor (NT), and the *URI*^{high} patients had higher *URI* levels in tumors than their paired non-tumor tissues, while the *URI*^{low} patients had lower tumoral *URI* levels than their non-tumor tissues (n=91 patients). **g,** Recurrence free survival rates and Overall survival rates were analyzed between *URI*^{high} group and *URI*^{low} group in Fudan_HCC_Cohort with mutant p53 (n=91 patients). **h,** Recurrence free survival rates and Overall survival rates were analyzed between *SCD1*^{high} group and *SCD1*^{low} group in Fudan_HCC_Cohort with mutant p53 (n=91 patients). Data are means ± SEM. HR: hazard ratio; Two-sided log-rank test (**a, b, d, e** and **g-h**) or two-tailed unpaired t-test (**f**) is used to determine the *P*-value. Source data are provided as a Source Data file.

Supplementary Figure 11

a

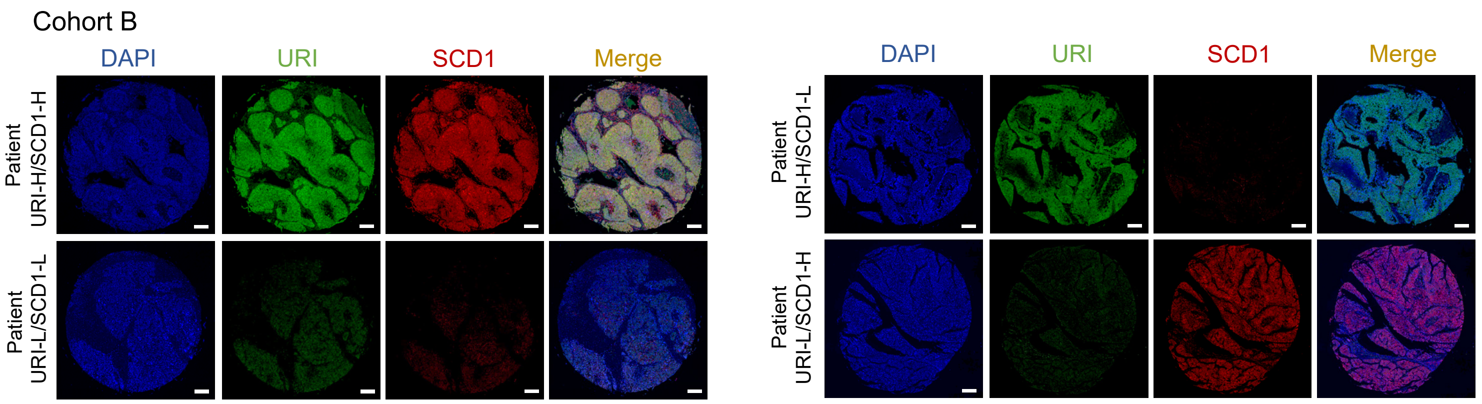

b

Cohort B		URI expression High (n=67)	URI expression Low (n=30)	P value	Person R
SCD1 expression	High (n=60)	55	5	<0.0001	0.600
	Low (n=37)	12	25		

c

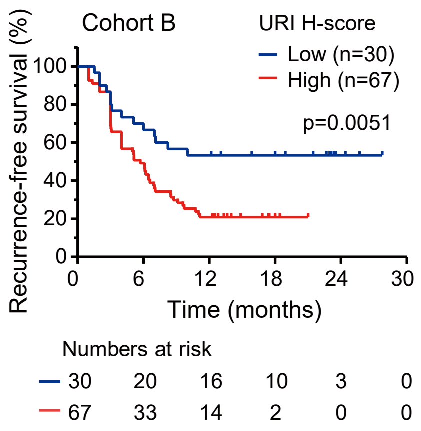

d

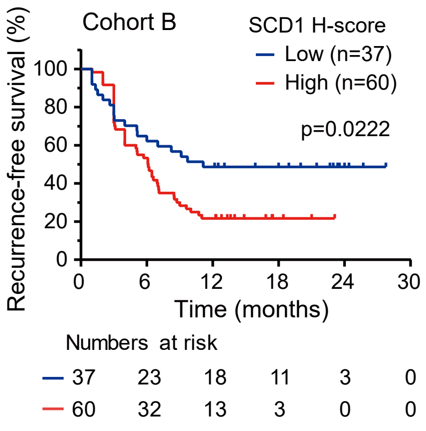

e

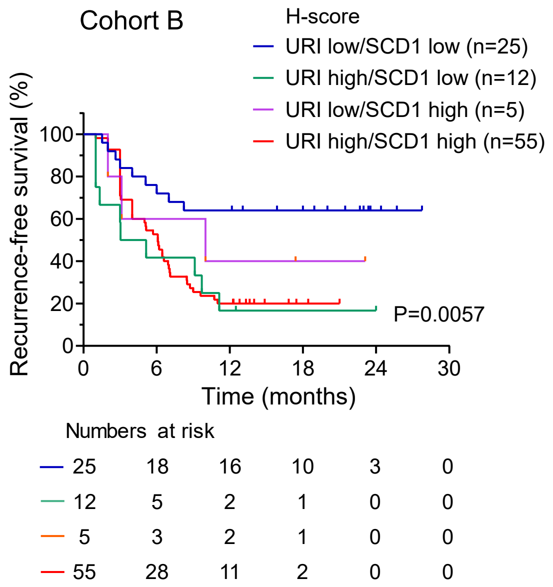

f

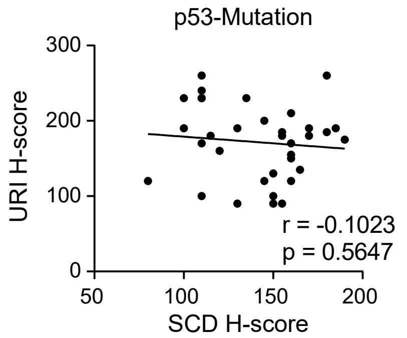

g

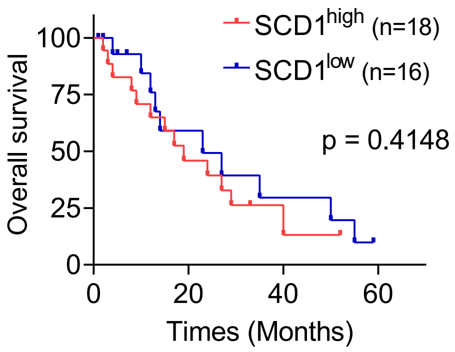

h

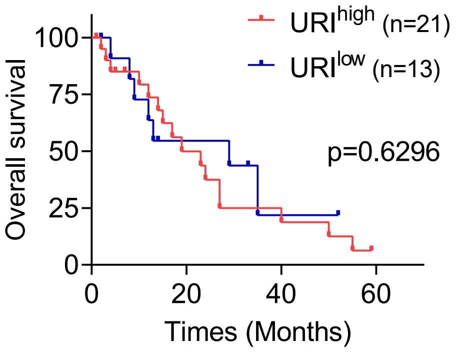

Supplementary Fig. 11: URI and SCD1 expression in HCC from cohort B and cohort C. a, Representative mIHC/IF images of URI (green) in combination with SCD1 (red) in HCC in cohort B (n=97 patients). Scale bar, 200 µm. **b,** Pearson's correlation between URI and SCD1 expression in HCC tissues according to the scores of immunohistochemistry staining (determined by two-tailed test) (n=97 patients). **c, d,** Recurrence free survival rates were analyzed between patients according to their URI H-score (**c**) or SCD1-H-score (**d**) in cohort B. Numbers below plots represent numbers of patients (n=97 patients). **e,** Recurrence free survival rates were analyzed among four subgroups (URI low/SCD1 low, URI high/SCD1 low, URI low/SCD1 high, URI high/SCD1 high). Numbers below plots represent numbers of patients (n=97 patients). **f,** Spearman's correlation between SCD1 H-score and URI H-score in cohort C with mutant p53 (determined by two-tailed test) (n=34 patients). **g**, **h**, Overall survival rates were analyzed according to tumoral SCD1 (**g**) or URI (**h**) levels in cohort C with mutant p53 (n=34 patients). Data are means ± SEM. Two-sided log-rank test (**c-e** and **g-h**) is used to determine the *P*-value. Source data are provided as a Source Data file.
