# Supplementary material for: URI alleviates tyrosine kinase inhibitors-induced ferroptosis by reprogramming lipid metabolism in p53 wild-type liver cancers
Source: Nat Commun. 2023 Oct 7;14:6269. doi: 10.1038/s41467-023-41852-z (PMC10560259; doi:10.1038/s41467-023-41852-z)
Supplement: Supplementary file 2 — Reporting Summary [file 41467_2023_41852_MOESM2_ESM.pdf]

nature portfolio

Corresponding author(s): Hongyang Wang, Liwei Dong, Lu Wang

Last updated by author(s): Sep 17, 2023

Reporting Summary

Nature Portfolio wishes to improve the reproducibility of the work that we publish. This form provides structure for consistency and transparency in reporting. For further information on Nature Portfolio policies, see our Editorial Policies and the Editorial Policy Checklist.

Statistics

For all statistical analyses, confirm that the following items are present in the figure legend, table legend, main text, or Methods section.

n/a	Confirmed	
☐	☒	The exact sample size (*n*) for each experimental group/condition, given as a discrete number and unit of measurement
☐	☒	A statement on whether measurements were taken from distinct samples or whether the same sample was measured repeatedly
☐	☒	The statistical test(s) used AND whether they are one- or two-sided<br>*Only common tests should be described solely by name; describe more complex techniques in the Methods section.*
☒	☐	A description of all covariates tested
☒	☐	A description of any assumptions or corrections, such as tests of normality and adjustment for multiple comparisons
☐	☒	A full description of the statistical parameters including central tendency (e.g. means) or other basic estimates (e.g. regression coefficient) AND variation (e.g. standard deviation) or associated estimates of uncertainty (e.g. confidence intervals)
☐	☒	For null hypothesis testing, the test statistic (e.g. *F*, *t*, *r*) with confidence intervals, effect sizes, degrees of freedom and *P* value noted<br>*Give P values as exact values whenever suitable.*
☒	☐	For Bayesian analysis, information on the choice of priors and Markov chain Monte Carlo settings
☒	☐	For hierarchical and complex designs, identification of the appropriate level for tests and full reporting of outcomes
☐	☒	Estimates of effect sizes (e.g. Cohen's *d*, Pearson's *r*), indicating how they were calculated

Our web collection on statistics for biologists contains articles on many of the points above.

Software and code

Policy information about availability of computer code

Data collection	Analyst 1.6.3 software MRM data acquisition mode (Applied Biosystems, Foster City, USA) was used for data collection of fatty acid profiling. MultiQuant (v3.0.2, Applied Biosystems, Foster City, USA) was used for data processing. The mIHC staining slides were scanned with 3DHISTECH. RNA-seq and CUT&Tag sequencing were done by Illumina NovaSeq 6000. Flow cytometry was performed by FACSVerse (BD Bioscience).
Data analysis	Statistical analysis was performed using GraphPad Prism 8 software (v8.4.2.679) and SPSS software (v26.0). Microscopic images were analyzed by ImageJ (v1.53e). GeneSet enrichement were performed by GSEA (v4.1.0). Data processing for CUT&Tag was performed using Fastp (v0.20.0), Bowtie2 (v1.3), SEACR (v1.3), DeepTools (v2.27.1), R package ChIPseeker (v1.12.1) and MEMEChIP (v5.0.5). RNA-seq data were analyzed by R package (v3.6.2). Flow cytometry data were analyzed by FlowJo (v10.0)

For manuscripts utilizing custom algorithms or software that are central to the research but not yet described in published literature, software must be made available to editors and reviewers. We strongly encourage code deposition in a community repository (e.g. GitHub). See the Nature Portfolio guidelines for submitting code & software for further information.

Data

Policy information about availability of data

All manuscripts must include a data availability statement. This statement should provide the following information, where applicable:

- Accession codes, unique identifiers, or web links for publicly available datasets
- A description of any restrictions on data availability
- For clinical datasets or third party data, please ensure that the statement adheres to our policy

The source data for all box plots, bars, and line graphs as well as the original uncropped Western blots can be found in the online Source Data File. The data of CUT&Tag and RNA-seq of HCC cells generated in this study have been deposited in the National Genomics Data Center under accession code HRA003798 for CUT&Tag (https://ngdc.cncb.ac.cn/search/?dbId=hra&q=HRA003798) and HRA003799 for RNA-seq (https://ngdc.cncb.ac.cn/search/?dbId=hra&q=HRA003799). The mass spectrometry proteomics data have been deposited to the ProteomeXchange Consortium via the PRIDE54 partner repository with the dataset identifier PXD045407 (http://www.ebi.ac.uk/pride/archive/projects/PXD045407). The publicly available data of WES and transcriptome sequencing of Fudan_HCC_Cohort used in this study can be viewed in NODE by accession OEP000321 (https://www.biosino.org/node/project/detail/OEP000321). The publicly available sequence data of Sorafenib-HCC cohort (Cohort C) used in this study can be viewed in NGDC with the accession number CRA001003 (https://ngdc.cncb.ac.cn/search/?dbId=bioproject&q=CRA001003&page=1). The lipidomic data have been deposited in figshare (DOI: 10.6084/m9.figshare.23690010). The public datasets of GSE96793 (https://www.ncbi.nlm.nih.gov/geo/query/acc.cgi?acc=GSE96793), GSE96794 (https://www.ncbi.nlm.nih.gov/geo/query/acc.cgi?acc=GSE96794) and GSE121153 (https://www.ncbi.nlm.nih.gov/geo/query/acc.cgi?acc=GSE121153) were employed.

Human research participants

Policy information about studies involving human research participants and Sex and Gender in Research.

Reporting on sex and gender	Sex and gender were not considered in the study design and determined based on patient medical records. We analyzed 134 patients (118 males, 16 females) in Cohort A and 97 patients (89 males, 8 females) in Cohort B in our study. In the previous reported sorafenib-treatment Cohort C, total 80 patients (72 males, 8 females) were finally enrolled. In HCC_Fudan_cohort about 80.6% of patients were males and 19.4% of patients were females. The distribution approximately resembles the ratio of males and females for HCC prevalence in the population of China. No stratified analyses based on genders were performed among these cohorts.
Population characteristics	The 134 patients with advanced HCC In cohort A (118 males, 16 females) are from Eastern Hepatobiliary Surgery Hospital (EHBH, Shanghai, China) with median age of 48 (range 28-73) and treated with hepatectomy. All the 97 patients (89 males, 8 females) in cohort B are from EHBH with median age of 46 (range 17-74), and these patients were at high risk of recurrence and received sorafenib therapy after hepatectomy. The detailed clinical characteristics of Cohort A and Cohort B are provided in our Supplementary Table 4 and Supplementary Table 5, respectively.<br>In cohort C, as mentioned in its related published article (doi: 10.7150/thno.41616), the HCC patients diagnosed for the first time at EHBH and underwent hepatectomy during 2006-2013, and 119 patients whom relapsed after the surgery and not suitable for the second operation were enrolled. The enrolled patients received mono sorafenib therapy or systemic therapy containing sorafenib according to NCCN guidelines for advanced hepatobiliary cancers. The collected specimens were analyzed by targeted exome sequencing. After the default quality control and a modest filter (coverage > 150 and broadness > 60% for the targeted regions), together with excluded samples with the abnormal high mutation rates, total 80 patients (72 males, 8 females) were left with median age of 51 (range 43-57). The detailed clinical characteristics and mutation landscapes can be viewed in the published article and the dataset CRA001003 in NGDC database. The mutation status of TP53 is provided in Supplementary Table 6.<br>In HCC_Fudan_Cohort, the detailed clinical characteristics of patients can be viewed in the published article (doi: 10.1016/j.cell.2019.08.052). Briefly, the patients were initially enrolled for CPTAC project (CHCC-HBV patients) and underwent primary curative resection from 2010 to 2014 at Zhongshan Hospital, Shanghai. The paired tumor, adjacent non-tumor liver tissues and blood samples were collected according to the CPTAC clinical sample collection procedures and the RNA-seq and WES date were collected. The patients with both WES and RNA-seq date were finally enrolled, and the patients with OS and RFS were then used for K-M survival analysis. The median age was 54 (range 20-79) and 80.6% of patients were males and 19.4% of patients were females.
Recruitment	No patients were recruited specifically for this study.
Ethics oversight	The procedure of human specimen collection was approved by the Ethics Committee of EHBH.

Note that full information on the approval of the study protocol must also be provided in the manuscript.

Field-specific reporting

Please select the one below that is the best fit for your research. If you are not sure, read the appropriate sections before making your selection.

[x] Life sciences [ ] Behavioural & social sciences [ ] Ecological, evolutionary & environmental sciences

For a reference copy of the document with all sections, see nature.com/documents/nr-reporting-summary-flat.pdf

Life sciences study design

All studies must disclose on these points even when the disclosure is negative.

Sample size	No statistical methods were used to predetermine sample sizes. Sample sizes were selected empirically from our previous experimental experience (PMID: 27091842; PMID: 31541481; PMID: 32967970; PMID: 36436593) or based on availability of samples with sufficient sequencing quality and clinical information.
Data exclusions	No data were excluded.
Replication	Except for the animal studies (one time), RNA-seq (one time) and Lipidomics (one time), each experiment was repeated at least three times.
Randomization	For cell experiments, all cells in each experiment were from the same pool of parental cells. For mouse experiment, mice were randomly assigned to different treatment groups.<br>For the human patients research, the enrolled patients in every cohort were received the same therapeutic regimen, respectively. Allocating patients into different experimental groups was not required.
Blinding	Due to the absence of experimental groups, there was no blinding in this study.

Reporting for specific materials, systems and methods

We require information from authors about some types of materials, experimental systems and methods used in many studies. Here, indicate whether each material, system or method listed is relevant to your study. If you are not sure if a list item applies to your research, read the appropriate section before selecting a response.

Materials & experimental systems

n/a	Involved in the study	
☐	☒	Antibodies
☐	☒	Eukaryotic cell lines
☒	☐	Palaeontology and archaeology
☐	☒	Animals and other organisms
☒	☐	Clinical data
☒	☐	Dual use research of concern

Methods

n/a	Involved in the study	
☒	☐	ChIP-seq
☐	☒	Flow cytometry
☒	☐	MRI-based neuroimaging

Antibodies

Antibodies used	The antibodies used in this studies were all listed in Supplementary table 7.<br>Antibodies used for immunoblotting:<br>Antibody (dilution, Company and Cat number)<br>URI (1:1000, Proteintech, 11277-1-AP), SREBP1 (1:200, Santa Cruz, sc-13551), SCD1 (1:1000, ABclonal, A16429), FASN (1:1000, ABclonal, A19050), FADS2 (1:1000, ABclonal, A10270), GPX4 (1:1000, ABclonal, A11243), GCLM (1:1000, ABclonal, A11444), GCLC (1:1000, ABclonal, A4499), SLC7A11 (1:1000, Proteintech, 26864-1-AP), ACSL4 (1:1000, Proteintech, 22401-1-AP), MDM2 (1:1000, ABclonal, A13327), Myc-Tag (1:1000, ABclonal, AE010), Flag-Tag (1:1000, ABclonal, AE005), His-Tag (1:1000, ABclonal, AE003), HA-Tag (1:1000, ABclonal, AE008), ACC (1:1000, CST, 3676), TRIM28 (1:1000, Proteintech, 15202-1-AP), p53 (1:1000, Proteintech, 10442-1-AP), p21 (1:1000, Proteintech, 10355-1-AP), USP5 (1:1000, ABclonal, A4202), USP7 (1:1000, ABclonal, A13564), USP14 (1:1000, ABclonal, A19589), Actin (1:5000, Proteintech, 66009-1-ig), GAPDH (1:5000, ABclonal, AC001).<br><br>Antibodies used for immunoprecipitation:<br>p53(Santa Cruz, sc-126), MDM2 (Santa Cruz, sc-965), Flag-Tag (CST, 8146) with 2-4 μg per 500 μg protein.<br><br>Antibodies used for ChIP:<br>SREBP1 (Santa Cruz, sc-13551), p53 (Santa Cruz, sc-126).<br><br>Antibodies used for IHC, mIHC/IF:<br>URI (1:200, Proteintech, 11277-1-AP), SCD1 (1:200, Abcam, ab236868), p53 (1:100, Santa Cruz, sc-126), Ki67 (1:1000, Abcam, ab15580).
Validation	All antibodies were purchased from the above stated companies.<br>Antibodies used for immunoblotting:<br>Antibody (dilution, Company and Cat number)<br>URI (1:1000, Proteintech, 11277-1-AP), https://www.ptgcn.com/products/RMP-Antibody-11277-1-AP.htm<br>SREBP1 (1:200, Santa Cruz, sc-13551), https://www.scbt.com/p/srebp-1-antibody-2a4?requestFrom=search<br>SCD1 (1:1000, ABclonal, A16429), https://abclonal.com.cn/catalog/A16429

FASN (1:1000, ABclonal, A19050), https://abclonal.com.cn/catalog/A19050
FADS2 (1:1000, ABclonal, A10270), https://abclonal.com.cn/catalog/A10270
GPX4 (1:1000, ABclonal, A11243), https://abclonal.com.cn/catalog/A11243
GCLM (1:1000, ABclonal, A11444), https://abclonal.com.cn/catalog/A11444
GCLC (1:1000, ABclonal, A4499), https://abclonal.com.cn/catalog/A4499
SLC7A11 (1:1000, Proteintech, 26864-1-AP), https://www.ptgcn.com/products/xCT-Antibody-26864-1-AP.htm
ACSL4 (1:1000, Proteintech, 22401-1-AP), https://www.ptgcn.com/products/ACSL4-Antibody-22401-1-AP.htm
MDM2 (1:1000, ABclonal, A13327), https://abclonal.com.cn/catalog/A13327
Myc-Tag (1:1000, ABclonal, AE010), https://abclonal.com.cn/catalog/AE010
Flag-Tag (1:1000, ABclonal, AE005), https://abclonal.com.cn/catalog/AE005
His-Tag (1:1000, ABclonal, AE003), https://abclonal.com.cn/catalog/AE003
HA-Tag (1:1000, ABclonal, AE008), https://abclonal.com.cn/catalog/AE008
ACC (1:1000, CST, 3676), https://www.cellsignal.cn/products/primary-antibodies/acetyl-coa-carboxylase-c83b10-rabbit-mab/3676?site-search-type=Products&N=4294956287&Ntt=3676&fromPage=plp&_requestid=11682197
TRIM28 (1:1000, Proteintech, 15202-1-AP), https://www.ptgcn.com/products/TRIM28-Antibody-15202-1-AP.htm
p53 (1:1000, Proteintech, 10442-1-AP), https://www.ptgcn.com/products/P53-Antibody-10442-1-AP.htm
p21 (1:1000, Proteintech, 10355-1-AP), https://www.ptgcn.com/products/P21-Antibody-10355-1-AP.htm
USP5 (1:1000, ABclonal, A4202), https://abclonal.com.cn/catalog/A4202
USP7 (1:1000, ABclonal, A13564), https://abclonal.com.cn/catalog/A13564
USP14 (1:1000, ABclonal, A19589), https://abclonal.com.cn/catalog/A19589
Actin (1:5000, Proteintech, 66009-1-ig), https://www.ptgcn.com/products/Pan-Actin-Antibody-66009-1-Ig.htm
GAPDH (1:5000, ABclonal, AC001), https://abclonal.com.cn/catalog/AC001

Antibodies used for immunoprecipitation:
p53(Santa Cruz, sc-126), https://www.scbt.com/p/p53-antibody-do-1?requestFrom=search
MDM2 (Santa Cruz, sc-965), https://www.scbt.com/p/mdm2-antibody-smp14?requestFrom=search
Flag-Tag (CST, 8146). https://www.cellsignal.cn/products/primary-antibodies/dykddddk-tag-9a3-mouse-mab-binds-to-same-epitope-as-sigma-s-anti-flag-m2-antibody/8146?site-search-type=Products&N=4294956287&Ntt=8146&fromPage=plp&_requestid=11682398

Antibodies used for ChIP:
SREBP1 (Santa Cruz, sc-13551), https://www.scbt.com/p/srebp-1-antibody-2a4?requestFrom=search
p53 (Santa Cruz, sc-126), https://www.scbt.com/p/p53-antibody-do-1?requestFrom=search

Antibodies used for IHC, mIHC/IF:
URI (1:200, Proteintech, 11277-1-AP), https://www.ptgcn.com/products/RMP-Antibody-11277-1-AP.htm

SCD1 (1:200, Abcam, ab236868), https://www.abcam.cn/scd1-antibody-epr21963-ab236868.html
p53 (1:100, Santa Cruz, sc-126), https://www.scbt.com/p/p53-antibody-do-1?requestFrom=search
Ki67 (1:1000, Abcam, ab15580). https://www.abcam.cn/ki67-antibody-ab15580.html

Eukaryotic cell lines

Policy information about cell lines and Sex and Gender in Research

Cell line source(s)	HepG2 (p53-wild), Hep3B (p53-null), Huh7 (p53-Y220C), HCT116 (p53-wild), HT29 (p53-R273H) and PLC/PRF/5 (p53-R249S) cells were purchased from Shanghai Cell resource center of Chinese Academy of Sciences. JHH1 (p53-wild) cells were kindly provided by Haojie Jin at the Shanghai Cancer Institute. HEK293T cells were purchased from the American Type Culture Collection (ATCC).
Authentication	All of the cell lines expect JHH-1 are maintained by the supplier and no additional authentication was performed. Short tandem repeat (STR) profiling of JHH-1 cell was tested.
Mycoplasma contamination	All cell lines were confirmed to be mycoplasma free with a mycoplasma detection kit and treated with Mycoplasma Elimination Reagent for the prevention of mycoplasma contamination.
Commonly misidentified lines (See ICLAC register)	none

Animals and other research organisms

Policy information about studies involving animals; ARRIVE guidelines recommended for reporting animal research, and Sex and Gender in Research

Laboratory animals	Pathogen-free male athymic nude mice (6-week-old, D000521) were purchased from the GemPharmatech Co., Ltd (Jiangsu, China). All the animals were housed in a specific pathogen-free (SPF) environment on a 12h light/dark cycle at temperature 20-25℃ and humidity 50-60% at the National Center for Liver Cancer, Shanghai, China.
Wild animals	No wild animals were used.

Reporting on sex	Experiments were performed in male nude mice to achieve successfully tumor xenograft models.
Field-collected samples	No field-collected samples were used.
Ethics oversight	All mice were treated according to protocols approved by the Naval Medical University Animal Care Facility and the National Institutes of Health guidelines.

Note that full information on the approval of the study protocol must also be provided in the manuscript.

Flow Cytometry

Plots

Confirm that:

- [x] The axis labels state the marker and fluorochrome used (e.g. CD4-FITC).
- [x] The axis scales are clearly visible. Include numbers along axes only for bottom left plot of group (a 'group' is an analysis of identical markers).
- [x] All plots are contour plots with outliers or pseudocolor plots.
- [x] A numerical value for number of cells or percentage (with statistics) is provided.

Methodology

Sample preparation	Cells (30000-50000 cells/well) were seeded in 6-well plates and treated with drugs for 48h. For tumor cell death detection, cells were resuspended in phosphate buffer saline (PBS) and incubated with PropidiumIodide (PI) (Sigma-Aldrich, P4170) for 30 minutes. For lipid peroxidation assay, cells were resuspended in PBS containing 20mM C11-BODIPY 581/591 (ABclonal, RM02821) and incubated for 1h at 37℃ in a cell culture incubator. The signals from both non-oxidized C11 (wave length > 580 nm) and oxidized C11 (wave length 505-550 nm) were monitored. The Liperfluo (Dojindo, L248) was used to evaluate cell lipid peroxidation according to the manufacturer's instructions. Cells were treated with drugs for 48h and incubated with 5 µM Liperfluo for 30min at 37°C and then detected by flow cytometry at 488nm excitation and 550nm emission.
Instrument	Cells were performed on LSRFortessa X-20 flow cytometer (BD Bioscience)
Software	Data analysis was performed using FlowJo V10.
Cell population abundance	At least 5,000 cells were analyzed for each sample.
Gating strategy	Cells were gated with foeward (FSC-A) and side (SSC-A) scatters to exclude debris, then gated with FSC-W and FSC-A to obtain single cell population. The cells receiving same drug treatment but without C11-BODIPY 581/591, PI or Liperfluo staining were measured as negative control.

- [x] Tick this box to confirm that a figure exemplifying the gating strategy is provided in the Supplementary Information.
