# Supplementary material for: URI alleviates tyrosine kinase inhibitors-induced ferroptosis by reprogramming lipid metabolism in p53 wild-type liver cancers
Source: Nat Commun. 2023 Oct 7;14:6269. doi: 10.1038/s41467-023-41852-z (PMC10560259; doi:10.1038/s41467-023-41852-z)
Supplement: Supplementary file 3 — Description of Additional Supplementary Files [file 41467_2023_41852_MOESM3_ESM.pdf]

Title: Supplementary Data 1.

Description: The RNA-seq data of HepG2-shURI and HepG2-Ctrl cells.

Title: Supplementary Data 2.

Description: The predicated binding sites of SREBP or p53 in the promoter region of *SCD1*.

Title: Supplementary Data 3.

Description: The list of potential URI-binding proteins in HEK-293 cells transfected with His-URI plasmid. The identified proteins from five repetitive experiments were shown.

Title: Supplementary Data 4.

Description: The clinical information of the cohort A.

Title: Supplementary Data 5.

Description: The clinical information of the cohort B.

Title: Supplementary Data 6.

Description: *TP53* mutations in sorafenib treated HCC patients from the cohort C.

Title: Supplementary Data 7.

Description: The detail information of the reagents used.

Title: Supplementary Data 8.

Description: The list of primers and oligos.
